# Reduction in weight and BMI and changes in Co-morbidities following laparoscopic adjustable gastric banding procedure for morbidly obese patients in Bahrain: a five year longitudinal study

**DOI:** 10.1186/2193-1801-2-19

**Published:** 2013-01-22

**Authors:** Khalid Al Khalifa, Claudio Violato, Ahmed Al Ansari

**Affiliations:** 1 FRCSI, Department of General Surgery, Bahrain Defense Force Hospital, West Riffa, Bahrain; 2 Medical Education Research Unit, Department of Community Health Sciences, University of Calgary, Calgary, Alberta Canada; 3 MBBCH, MRCSI, MHPE, Department of General Surgery, Bahrain Defense Force Hospital, West Riffa, Bahrain; 4 MBBCh, MRCSI, MHPE, University Ambrosiana and University of Calgary, Faculty of Medicine, 3330 Hospital Drive NW, Calgary, AB T2N 1N4 Canada

**Keywords:** Bariatric surgery, Adjustable gastric band, Excess weight loss

## Abstract

**Background:**

Obesity and its related illnesses are pan-endemic health problems which require intervention. Laparoscopic Adjustable Gastric Banding (LAGB) is seen as a safe surgical procedure with satisfactory results on weight reduction and improvement in obesity related illness.

**Methods:**

Data were collected in a repeated-measures longitudinal five year study for 143 morbidly obese patients who underwent laparoscopic adjustable gastric banding (LAGB). Follow up was continued from 3 to 60 months post operatively. Patients were assessed for diabetes, hypertension and dyslipidemia.

**Results:**

Repeated measures ANOVAs revealed that both men and women lose weight and reduce their BMIs at a consistent rate. At 3, 6 and 9 months post-operative there are no differences in percent weight loss between men and women with mean weight loss at 8.9%, 13.1% and 16.0% respectively of pre-operative weight. At 12, 24 and 60 months post-operatively, however, men significantly increase the percentage of weight loss as well as improve their BMI compared to women (p < .05). At 24 months post-operative, men and women have BMIs of 36.03 and 32.85, both still in the obese range. By 60 months men have achieved a BMI that is slightly under the obese range into the overweight range (30.76) while women (BMI = 36.61) were still in the obese range. At 60 months, men have lost a total of 33.75% of their pre-operative body weight while women have lost a total of 21.50. Diabetes, hypertension and dyslipidemia were significantly reduced in the sample post-operatively (p < .01).

**Conclusion:**

LAGB is a safe and effective surgical procedure for morbidly obese patients resulting in weight loss, BMI decrease and reduction in co-morbid illnesses.

## Introduction

Obesity is recognized as an international health problem in both developed and developing societies. According to the World Health Organization (WHO), the prevalence of obesity in Bahrain was put at 21.2% and 35.2% among males and females respectively. The mean body mass index (BMI) increased dramatically from 24 kg/m^2^ to 28 kg/m^2^ and from 25.6 kg/m^2^ to 29.3 kg/m^2^ for male and female respectively between the periods of 1980 to 2008 (World Health Organization 2011 ). In the USA, it is estimated that over 130 million adults are overweight or obese. Obesity is now considered to be the most common cause of death, resulting in 300,000 deaths annually, approximately 14% of all deaths (World Health Organization 2011 ). Once an individual becomes obese, the risk of developing a co-morbid illness such as diabetes mellitus (DM), hypertension (HTN), and hyperlipidemia increase significantly (Wang et al. 2008 ). The risk of type 2 diabetes mellitus (T2DM), for example, has been shown to increase approximately 40-fold as BMI increases from <23 kg/m^2^ to> 35 kg/m^2^. ( Pontiroli 2008 ).

The WHO has recognized the impact of obesity on health and quality of life and thus recommended that studies of health changes associated with weight loss should be a research priority (Leonardo et al. 1997 ). Other Organizations such as the National Institutes of Health, and surgeons in general, recognize the severity of obesity and its related co-morbid illnesses (Dixon & O’ Brien 2002 ). They also acknowledge the impact of obesity on health, functioning, and well-being (Karmali et al. 2011 ). This has resulted in a worldwide increase in bariatric surgery (Ahroni et al. 2005 ).

Bariatric surgery is a common treatment for morbid obesity. Currently the two most common surgical procedures are laparoscopic adjustable gastric banding (LAGB) and laparoscopic Roux-en Y gastric bypass (LRYGBP). LRYGBP is the most common bariatric surgery in the USA whereas in Europe and Australia, LAGB is more common (Korenkov et al. 2007 ). Laparoscopic adjustable gastric banding (LAGB) is seen as a simple and safe surgical procedure in individuals with morbid obesity, with satisfactory weight loss, improvement in co-morbidities and significant improvement in quality-of-life (Korenkov et al. 2007 ).

Weight loss post bariatric surgery including laparoscopic adjustable gastric banding seems to have a major impact on obesity and its related illnesses (Kinzl et al. 2007 ). Further research, especially long-term follow-up studies is required to assess the effectiveness and safety of LAGB. Moreover, further research of under-studied populations such as in the Middle East is required for LAGB. The major purpose of the present study, therefore, was to evaluate the impact of laparoscopic adjustable gastric banding on weight reduction, BMI decrease and obesity related illnesses such as diabetes, hypertension, and hyperlipedimia in patients in Bahrain in a 5 year follow-up.

## Methods

### Patients

One hundred and forty-three patients (107 women - 74.8% / 36 men - 25.2%; mean age = 29.9, SD=7.16) underwent LAGB between June 2003 and February 2010. Patient selection was based on the criteria that has been identified by the 1991 NIH Consensus Conference Statement on Gastrointestinal Surgery for Severe Obesity (Brancatisano et al. 2007 ).

The mean weight pre-operative for women was 118.74 kg with BMI = 45.75 and for men was 137.51 kg with BMI = 45.30. Patients were assessed for co-morbidities, operative time, operative complication, hospital stay, and follow up at 3, 6, 9, 12, 24 and 60 months. The mean operative time was 70.2 minutes (SD = 23.5). There were no major complications or patient mortality during the study period. All patients had undergone various conservative approaches (e.g., diet changes) for morbid obesity over a period of twelve or more months without any effect.

### Procedures

Surgical technique - All of the LAGB surgeries were performed by a single gastro-intestinal (GI) surgeon (KA) in one hospital (Bahrain Defense Force Hospital). The procedure involved insertion of four to five trocars in the upper abdomen. The band (Swiss adjustable band) was placed around the fundus of the stomach straight away below the esophagogastric junction via a retrogastric tunnel created using the pars flaccid approach. In some cases, the band was fixed by two or three anterior imprecating gastro-gastric sutures.

Postoperative first visit - The initial follow up visit was at one week postoperatively. Reviews continued monthly for the first year, and then reduced to a visit every two months in the following years. All adjustments to the band were performed with 7–8 ml of contrast under fluoroscopic imaging.

Co-morbid disease - Patients with the following co-morbidity (Type 2 Diabetes, Dyslipidemia, and Hypertension) were identified. For each patient suffering from diabetes, fasting blood sugar and Hba1c was recorded. Patients with dyslipidemia, cholesterol level and low density lipoprotein (LDL) were measured. All hypertensive patients were evaluated according to their blood pressure measurement and medication dosage pre and postoperatively. Co-morbid illness was considered resolved if the patient no longer required medication after surgery and specific laboratory values returned to normal. Improvement of co-morbidity was defined by dose reduction in medication with improvement in specific laboratory values. Treatment of co-morbid conditions was supervised by the relevant specialist.

Operative complication - Intra-operative and post operative complications were recorded. Complications in the form of intra-operative bleeding, band intolerance, and wound infection were recorded. No gastric perforation, necrosis, or band slippages were detected.

### Data analyses

Data analyses were conducted with SPSS version 19.0. In addition to descriptive statistics, a two-way repeated measures analysis of variance (2-way rep-ANOVA) employing sex (2 levels) and time (7 levels) as independent variables and BMI as the dependent variable was conducted. A second 2-way rep-ANOVA employing sex (2 levels) and time (7 levels) as independent variables and weight (kg) as the dependent variable was conducted. Changes in co-morbidities were analyzed with Fisher’s exact test. The level of significance was set at p < 0.05.

### Ethics

The study was approved by the Ethics Committee of Bahrain Defense Force Hospital. Each patient provided informed consent to take part in the study.

## Results

There was significant differences in the first 2-way rep-ANOVA (sex × time) with BMI as the dependent variable (Time: Wilk’s lambda = 0.156, p < .001; Sex × time: Wilk’s lambda = 0.922, p < .05) as there was for weight (kg) as the dependent variable (Time: Wilk’s lambda = 0.030, p < .001; Sex × time: Wilk’s lambda = 0.833, p < .01). These results are summarized in Table 1 as are the results of percentage weight loss over the post-operative period.

**Table 1:** **BMI, weight and percent weight loss, percent of excess weight loss, compared for men and women pre-operative and six measurements post-operative**

Time		BMI		Weight (kg)		% weight loss	% EWL	
	Sex	Mean	SD	Mean	SD			n (total 143)
Pre-operative	Women	45.75	6.71	118.74	16.90	------	------	107
	Men	45.30	5.77	137.51	19.44	------	------	36
3 months post-operative	Women	42.01	6.59	108.81	17.39	8.39	17.65	107
	Men	40.54	5.07	123.40	18.71	10.28	19.07	36
6 months post-operative	Women	40.17	6.50	103.90	17.28	12.49*	26.30*	107
	Men	38.39	5.09	116.80	18.60	15.09	27.90	36
9 months post-operative	Women	38.88	6.42	100.47	16.81	15.38	32.48	107
	Men	36.65	4.82	112.95	20.74	17.94	33.10	36
12 months post-operative	Women	37.54*	6.59	97.11	17.13	18.20*	38.47*	107
	Men	34.78	4.81	105.86	18.08	22.98	42.70	36
24 months post-operative	Women	36.03*	6.84	92.99	17.65	21.69*	45.70*	107
	Men	32.85	4.60	100.22	17.32	26.85	50.30	36
60 months post-operative	Women	36.61*	8.69	94.58	21.66	21.50*	42.95*	39
	Men	30.76	4.85	93.27	19.81	33.75	59.00	13

* p < .05; ** p < .01.

**Figure 1:**
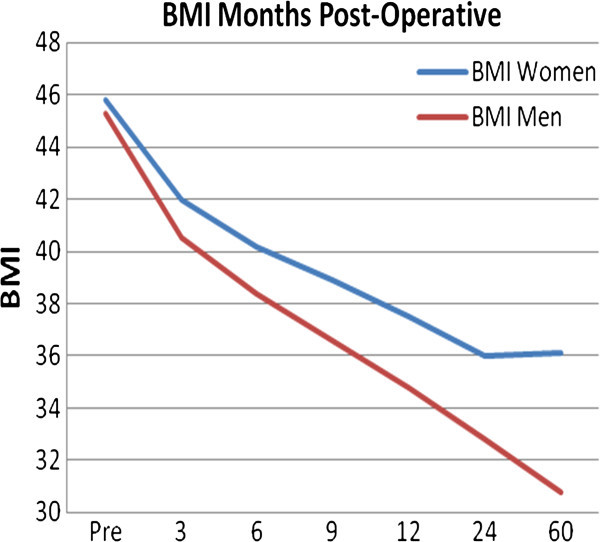
**BMI for men and women assessed at months post-operative.**

Additionally, the change in BMI from pre-operative to post-operative periods is represented graphically in Figure 1. A close inspection of Table 1 and Figure 1 reveals that both men and women lose weight and reduce their BMIs at a consistent rate. At 3, 6 and 9 months post-operative there are no differences in percent weight loss between men and women with mean weight loss at 8.9%, 13.1% and 16.0% respectively of pre-operative weight and mean % of (EWL) of 17.65%, 27.9%, and 33.1% (Table 1). At 12, 24 and 60 months post-operatively, however, men significantly (p < .05) increase the percentage of weight loss as well as improved their BMI compared to women. This is also evident from Figure 1. At 24 months post-operative, men and women have BMIs of 36.03 and 32.85 with EWL 45.7% and 50.3% respectively, both still in the obese range. Although the sample has had considerable attrition by 60 months, men have achieved a BMI (30.76) that is marginally outside the obese range into the overweight range while women (BMI = 36.61) are still in the obese range. At 60 months, men have lost a total of 33.75% of their pre-operative body weight which is equivalent to 59% of (EWL), while women have lost a total of 21.50% which is representing 42.95% of (EWL).

Analyses of the co-morbidities (at 24 months) indicated that the majority of diabetics pre-operative (n=18) were non-diabetic post-operative (n=2 diabetics; Chi square = 94.6, Fisher’s exact test < .001). Similarly, 19 patients had hyperlipidemia pre-operatively but this number was reduced to 6 post-operatively (Chi square = 73.6, Fisher’s exact test < .001). Finally, 14 patients had hypertension pre-operatively and this was reduced to 5 patients post-operatively (Chi square = 96.1, Fisher’s exact test < .001). There was, therefore, a significant reduction in all three co-morbidities that were assessed.

## Discussion

This is the first study with a five year follow-up of LAGB in Bahrain. It confirms that LAGB is a safe and effective surgery with a good result in weight reduction and changes in co-morbid illnesses. It also confirms that LAGB can be performed with a minimum of significant complications, which has been confirmed by other studies (1991).

### Weight loss

Weight reduction is the most commonly reported outcome measure of bariatric surgery (Frigg et al. 2004 ). Bariatric surgery induces a significant loss of fat mass (FM), and in particular of visceral fat, more than of subcutaneous (Koba et al. 2000 ). However, weight loss after LAGB with an average excess weight loss of 50-60% is less than after gastric bypass or biliopancreatic diversion ( Pontiroli 2008 ). In the present study, at 60 months, men have lost a total of 33.75% of their pre-operative body weight which is 59% of EWL while women have lost a total of 21.50% which representing 42.96% of EWL. Although the sample has had considerable attrition by 60 months, men have achieved a BMI (30.76) that is marginally outside the obese range into the overweight range while women (BMI = 36.61) are still in the obese range. This sex difference requires further investigation as to why women remain in the obese range and did not continue to lose weight between the period of 24 and 60 months respectively. We speculate that cultural differences (e.g., higher acceptance of obesity in women than in men) may play a role these results. Moreover, pregnancy as well may have contributed to the decreased weight loss for women which were reported between the periods of two to five years respectively in this study. Other studies have reported unexpected pregnancies in previously infertile women due to weight loss (Weiss et al. 2001 ). The effect of weight loss on obesity-related co-morbidities following LAGB surgery is also a decisive criterion for success in bariatric surgery in addition to weight loss and BMI reduction ( Dargent 2004 ; Frigg et al. 2004 ).

### Diabetes mellitus

LAGB has a dramatic effect on T2DM due to weight loss induced by gastric banding. This remarkable improvement in diabetes with weight loss, following LAGB surgery is related to the double effect of improved insulin sensitivity and pancreatic beta-cell function (Leonardo et al. 1997 ). Most gastric band studies have proved that between 66% and 96% of patients achieve resolution and /or improvement in terms of normalization of laboratory values and reduction/ or cessation of antidiabetes medication (Kinzl et al. 2007 ; Omana et al. 2010 ). Our results are consistent with these findings. Out of eighteen patients, 16 (88%) showed improvement in DM in the period of 2 to 5 years and two patients showed no improvement.

### Hypertension

The high prevalence of hypertension in the morbidly obese is one of the main risk factors for cardiovascular disease (Pontiroli et al. 2002 ). Reports confirm the reduction in both systolic and diastolic blood pressure following LAGB (Leonardo et al. 1997 ). Other studies have also shown resolution and/or improvement of hypertension ranging from 47.5% to 86% in obese patients (Kinzl et al. 2007 ; Omana et al. 2010 ). Prior to surgery, many patients remain hypertensive despite medical therapy. Several studies have now demonstrated that blood pressure is easier to be controlled post LAGB, and many patients are able to discontinue antihypertensive medication (Titi et al. 2007 ). In the present study the number of hypertensive patients were reduced from 14 to 5 patients post LAGB surgery representing an improvement in 68.2% of patients with hypertension.

### Dyslipidemia

Increased fasting triglyceride and decrease high-density lipoprotein (HDL) - cholesterol concentration and increased low-density lipoprotein (LDL) is highly atherogenic and the most common pattern associated with coronary disease (Bacci et al. 2002 ). Weight loss post LAGB is also associated with improvement in dyslipidemia in the form of reduction of triglyceride and low-density lipoprotein. Our result showed that the number of patients with hyperlipedemia reduced from 19 patients to six patients post-operatively in concordance with other studies that have reported improvement in lipid profile ranging from 63.5% to 95% (Kinzl et al. 2007 ).

## Conclusion

LAGB is an effective and safe procedure for weight loss in morbidly obese patients. After gastric banding, patients with morbid obesity experience a significant reduction in major co-morbid illness. The benefit of sustained significant weight reduction following LAGB surgery on the serious side-effects of obesity was demonstrated by its impact on diabetes, hypertension and dyslipidemia. Therefore, the success of LAGB should be judged by the weight loss and the improvement of obesity-associated co-morbidities.

Notwithstanding the positive results, further research is required to explore sex differences in the results of LAGB, as well as other cross-cultural differences that may exist. Moreover, continued further longitudinal studies of LAGB will be useful to further evaluate the long term impact of this bariatric procedure. Meanwhile, the results of the present study indicate that LAGB is a safe and effective surgical procedure for morbidly obese patients resulting in weight loss, BMI decrease and reduction in co-morbid illnesses.
